# Comparison of Manual and Automated Measurements of Monodominant Follicle Diameter with Different Follicle Size in Infertile Patients

**DOI:** 10.1371/journal.pone.0077095

**Published:** 2013-10-10

**Authors:** Ping Pan, Xiaoli Chen, Yu Li, Qingxue Zhang, Xiaomiao Zhao, Madafeitom M. A. Bodombossou-Djobo, Dongzi Yang

**Affiliations:** 1 Department of Obstetrics and Gynecology, Memorial Hospital of Sun Yat-Sen University, Guangzhou, Guangdong, People’s Republic of China; 2 Department of Reproductive Medicine, Guangdong Provincial Family Planning Research Institute, Guangzhou, Guangdong, People’s Republic of China; 3 Fertility Medical Center, Guangzhou Elizabeth Women's Hospital, Guangzhou, Guangdong, People’s Republic of China; Bascom Palmer Eye Institute, University of Miami School of Medicine;, United States of America

## Abstract

This study evaluated the consistency of manual and automated measurements of monodominant follicle diameter with different follicle size in infertile patients. Transvaginal two-dimensional (2D) ultrasound and SonoAVC (Sonography-based Automated Volume Calculation) were both performed in 226 infertile patients with monodominant follicle growth. 2D diameters were separately compared with SonoAVC-generated d(V) and m-d values in different follicle category, i.e. >10 to 14 mm, >14 to 18 mm, >18 to 22 mm and >22 mm. There was moderate degree of consistency between 2D diameter and SonoAVC-generated parameters regardless of follicle size. The mean differences were 0.82 mm between 2D diameter and SonoAVC-generated d(V) value, and 0.22 mm between 2D diameter and SonoAVC-generated m-d value, respectively. The discrepancy of manual and automated measurements tended to increase as follicle size increased. Our study suggested that compared with manual measurement, SonoAVC might underestimate follicle size. The absolute size of a follicle affected the consistency of two techniques.

## Introduction

To date, transvaginal two-dimensional (2D) ultrasound has been widely used to track ovarian follicle growth, confirm ovulation and detect ovulatory disfunction. 2D follicular diameter is a common ultrasonic parameter for the assessment of follicle size, which is conventionally restricted to manual determination. More recently, the advent of SonoAVC (Sonography-based Automated Volume Calculation) has allowed us to assess follicle size automatically. SonoAVC can identify and quantify hypoechoic regions within a three-dimensional (3D) dataset, such as a follicle in an acquired ovarian volume, and provide automatic estimation of their dimensions [1].

Previous studies have demonstrated that follicular volume measured by SonoAVC is more accurate than that estimated from 2D ultrasound, and equivalent to the aspirated follicular fluid volume [2-5]. Besides follicular volume, SonoAVC as well as provides other ultrasonic parameters, such as the volume-based diameter d(V) and mean follicular diameter (m-d). It has been shown that SonoAVC-generated d(V) and m-d values correlated extremely well with the manual measurements by 2D ultrasound, and the observed differences were <1 mm. Yet, some studies used SonoAVC to determine multiple growing follicles in hyperstimulated ovaries, and evaluated follicles ranging in wide diameter from 2.3 to 32 mm, some studies did not list the particular size of the follicles [6,7]. 

SonoAVC provides the opportunity to apply volumetric measure to track follicle growth and assess follicle size automatically, however, the cutoff value of follicular volume as a new parameter needs to be established. As a result, the use of SonoAVC-generated d(V) and m-d values but not follicular volume as surrogates of 2D diameter is of clinical relevance in practice at present time. Moreover, the applicability of SonoAVC still requires further study, and it is not clear whether there would be a difference of the consistency of manual and automated measurements for dominant follicles with various size. To clarify this issue, in the present investigation we compared SonoAVC with 2D ultrasound for the assessment of a cohort of dominant follicles in various size category. Meanwhile, to avoid possible confounding effects of multiple developing follicles on the reliability of our study, we preferred to use the experimental model of monodominant follicle growth in infertile patients.

## Materials and Methods

### Subjects

The infertile patients undergoing infertility treatment in outpatient clinic, or artificial insemination and frozen-thawed embryo transfer in the reproductive centre of Memorial Hospital of Sun Yat-Sen University from July 2012 to November 2012 were recruited. Only natural and mildly stimulated cycles with pre-ovulatory monodominant follicle growth (>10 mm in diameter) were included in this study, and the cycles with two or more developing follicles >10 mm were excluded. The protocols of mildly ovarian stimulation were following: (a) clomiphene citrate with a dosage of 50 mg/day from day 3 to day 7; (b) letrazole with a dosage of 5 mg/day from day 3 to day 7. A total of 226 infertile patients contributed to this study. The study was approved by the Human Research and Ethics Committee of Sun Yat-Sen Memorial Hospital, and all patients gave written informed consent.

### 2D follicular diameter measurement

A transvaginal scan was performed using a General Electric Voluson E8 Expert instrument (GE Medical Systems, Zipf, Austria) equipped with a 5 to 9 MHz transvaginal microconvex volume probe. All ultrasound examinations were performed by a single experienced investigator (Ping Pan) to avoid interobserver variation. By using the real-time 2D mode, two orthogonal diameters (d1 and d2) at the largest follicle plane were determined by placing calipers at the inner follicle border, as the standard clinical practice in our reproductive centre. The vaginal probe was placed to the follicle as close as possible, in order to make follicle borders visualized clearly. Each 2D follicular diameter was examined three times consecutively during the examination. Mean follicular diameter in each measurement corresponded to (d1 + d2)/2, then the average value from three measurements for each follicle was used for statistical analysis.

### SonoAVC scan

After completion of 2D measurement, SonoAVC was initiated to capture a 3D volume of each follicle, as reported in the literatures [1,7,8]. In brief, the sweep angle in 3D volume mode was set to 120° so as to include the entire ovary, and a 3D dataset was acquired using the high-quality, slow-sweep mode. Similarly, SonoAVC scan was also replicated for three times for each follicle. Post-processing of SonoAVC was performed with 4D View (version 10.3; GE Medical Systems) by the same investigator (Ping Pan) at least one month after data collection to prevent the recall of 2D examination. The settings of growth and separation within the software were kept uniform at default values of ‘mid’ for all follicle measurements. 

The ultrasonic parameters generated by SonoAVC included d(V), dx, dy, dz, m-d and V, but according to the study’s design, we only enrolled d(V), m-d and V for analysis. In detail, d(V) was the diameter of a perfect sphere with the same volume as the follicle, and m-d was the arithmetic mean of the three longest orthogonal diameters (dx, dy and dz), and V was the volume of the identified follicle based on the voxel count within it [7]. Accordingly the average values of all SonoAVC-generated parameters from three repeated measurements for each follicle were analyzed. 

### Statistical Analysis

Statistical analysis was performed by SPSS software (Statistical Package for Social Sciences, SPSS Inc, Chicago, IL, USA) version 13.0. Mean and SD were used for description of variables. Since it is hypothesized that the absolute size of a follicle might affect the consistency of manual and automated measurements, subgroup analysis was conducted based on the absolute follicular size. Consequently, all pre-ovulatory monodominant follicles were arbitrarily sorted into four groups based upon 2D follicular diameters, i.e. >10 to 14mm, >14 to 18mm, >18 to 22mm and >22mm, as these thresholds had clinical importance according to previous studies [7,9].

The intra-observer reliability of 2D measurement and SonoAVC was assessed by intraclass correlation coefficients (ICCs) and its 95% confidence intervals (CI) using a one-way random model, by calculating those 2D and SonoAVC-generated parameters obtained from three measurements for each subject. Correlation between 2D follicular diameter and SonoAVC-generated d(V), m-d and V measurements were separately evaluated using the Pearson correlation coefficient. The concordance for SonoAVC and manual 2D measurement was calculated by ICCs with absolute agreement and 95% CI using a two-way mixed model. The ICCs range from 0 to 1, and values under 0.10 are considered virtually none, 0.11-0.40 slight, 0.41-0.60 fair, 0.61-0.80 moderate, and 0.81-1.00 substantial [10]. Also, we used the limits of agreement method as reported by Bland and Altman [11]. The mean difference, the upper and lower limits of agreement were calculated and presented. The range between the upper and lower limits of agreement provides more information to judge how well two methods of measurement agree. The smaller the range between the upper and lower limits of agreement the better the agreement is [12]. P values of <.05 were considered significant.

## Results

### General characteristics

The mean age of participants was 32.21±4.25 years (range 23-44 years). Natural cycles accounted for 53.10% (120/226), and mildly stimulated cycles accounted for 46.90% (106/226). Of the participants, 53.10% (120/226) performed artificial insemination, 37.61% (85/226) performed frozen-thawed embryo transfer, and 9.29% (21/226) underwent infertility treatment in outpatient clinic. Two hundred and twenty-six pre-ovulatory monodominant follicles were studied in 226 patients, with 57.08% (129/226) from right ovary, and 42.92% (97/226) from left ovary. The number of ultrasonography per follicle in the menstrual cycle was 1 in 157 follicles, 2 in 52 follicles and 3 in 17 follicles, therefore 312 follicular data were enrolled in the analysis. Mean 2D follicular diameters were 18.09±3.37 mm (range10.50-27.33mm), mean SonoAVC-generated d(V) values were 17.26±3.41 mm (range 9.80-28.13 mm), mean SonoAVC-generated m-d values were 17.87±3.51 mm (range 10.43-28.50 mm), mean SonoAVC-generated follicular volumes were 3.01±1.75 mL (range 0.49-11.65 mL). 

### Intraobserver reliability of 2D measurement and SonoAVC

The ICCs were 0.986 (95% CI: 0.983-0.989) for 2D follicular diameter, 0.992 (95% CI: 0.990-0.993) for SonoAVC-generated d(V) value, and 0.993 (95% CI: 0.991-0.994) for SonoAVC-generated m-d value, suggesting good intraobserver reliability for each measurement.

### Correlation coefficient of 2D measurement and SonoAVC

On the whole, there were strong positive correlations between 2D follicular diameter and SonoAVC-generated parameters. The Pearson correlation coefficient was 0.960 for 2D diameter and SonoAVC-generated d(V) value, 0.958 for 2D diameter and SonoAVC-generated m-d value, and 0.929 for 2D diameter and SonoAVC-generated V value, respectively (P=0.000). When manual measurement and SonoAVC were separately compared in each follicle category, we found a moderate correlation between two techniques, with Pearson correlation coefficient ranging from 0.718 to 0.788. On the contrary, correlation coefficient between SonoAVC-generated d(V) and m-d value was still high, as presented in Table 1.

**Table 1:** Correlation coefficients of 2D follicular diameter and SonoAVC-generated parameters in various follicle category.

		Follicle size (mm)			
Ultrasonic parameters	Total (n=312)	>10 to 14 (n=46)	>14 to 18 (n=107)	>18 to 22 (n=118)	>22 (n=41)
2D diameter versus SonoAVC d(V)	0.960	0.775	0.788	0.740	0.743
2D diameter versus SonoAVC m-d	0.958	0.738	0.775	0.723	0.718
2D diameter versus SonoAVC V	0.929	0.744	0.773	0.745	0.762
SonoAVC d(V) versus SonoAVC m-d	0.995	0.974	0.980	0.971	0.974

### The ICCs for 2D measurement and SonoAVC

Accordingly the ICCs were 0.933 (95% CI: 0.710-0.972) for 2D diameter and SonoAVC-generated d(V) value, and 0.955 (95% CI: 0.943-0.965) for 2D diameter and SonoAVC-generated m-d value (P=0.000). Table 2 illustrated the ICCs of manual 2D measurement and SonoAVC in each follicle category, and we found moderate concordance between two techniques, with ICCs ranging from 0.585 to 0.742. Regardless of follicle size, 2D diameter versus SonoAVC-generated d(V) value corresponded to a lower ICCs value when compared with 2D diameter versus SonoAVC-generated m-d value.

**Table 2:** The ICCs of 2D measurement and SonoAVC in various follicle category.

		Follicle size (mm)			
Ultrasonic parameters	Total (n=312)	>10 to 14 (n=46)	>14 to 18 (n=107)	>18 to 22 (n=118)	>22 (n=41)
2D diameter versus SonoAVC d(V)					
ICCs*	0.933	0.602	0.665	0.587	0.585
95% confidence intervals	0.710-0.972	0.001-0.834	0.213-0.836	0.136-0.786	0.107-0.805
2D diameter versus SonoAVC m-d					
ICCs*	0.955	0.711	0.742	0.679	0.687
95% confidence intervals	0.943-0.965	0.519-0.832	0.642-0.817	0.567-0.766	0.487-0.819

* ICCs = intraclass correlation coefficients

### The limits of agreement between 2D measurement and SonoAVC

The mean differences were 0.82 mm between 2D diameter and SonoAVC-generated d(V) value, and 0.22 mm between 2D diameter and SonoAVC-generated m-d value, respectively. However, the observed differences in each follicle category between 2D diameter and SonoAVC-generated d(V) value increased as the follicle size increased, consequently the range of the limits of agreements were gradually wider in large follicle size as compared with in small follicle size. Unexpectedly, the observed differences in each follicle category between 2D diameter and SonoAVC-generated m-d value did not change substantially, but the range of the limits of agreements likewise appeared to be wider for large follicle size in comparison with small follicle size, shown in Table 3. 

**Table 3:** Bland-Altman plot analysis of 2D measurement and SonoAVC in various follicle category.

		Follicle size (mm)			
Ultrasonic parameters	Total (n=312)	>10 to 14 (n=46)	>14 to 18 (n=107)	>18 to 22 (n=118)	>22 (n=41)
2D diameter versus SonoAVC d(V)					
Mean difference	0.82±0.95	0.68±0.62	0.77±0.92	0.86±1.00	1.02±1.19
Upper limit of agreement	-1.08	1.92	2.61	2.86	3.40
Lower limit of agreement	2.72	-0.56	-1.07	-1.14	-1.36
2D diameter versus SonoAVC m-d					
Mean difference	0.22±1.01	0.24±0.66	0.20±1.00	0.21±1.07	0.26±1.18
Upper limit of agreement	-1.80	1.56	2.20	2.35	2.62
Lower limit of agreement	2.24	-1.08	-1.80	-1.93	-2.10

## Discussion

Whenever a novel technique comes out, comparison between the new and conventional method is indispensable. This study was unique in that we not only used different statistical tools to evaluate the concordance of 2D ultrasound and SonoAVC, but also compared two techniques in various follicle size category for further detail. Our data showed that there was moderate degree of consistency between 2D follicular diameter and SonoAVC-generated d(V) and m-d measurements regardless of follicle size, either analyzed by Pearson correlation coefficient or ICCs. The mean differences between 2D diameter and SonoAVC-generated parameters were <1 mm, and interestingly the discrepancy of two techniques tended to increase with the follicle size, revealed by the limits of agreement.

 The greatest strength of SonoAVC lies in accurate measurement of follicular volume, as mentioned above [2-5]. It is well known that a follicle is after all a three-dimensional structure, many investigators have already confirmed follicular volume is the most accurate measure of its size [2,3,7,13]. Because SonoAVC assessed follicular volume on the basis of the voxel count within it, this measurement seemed objective and reproducible [5]. Thus, SonoAVC had been considered to standardize the process of follicular assessment. What is more, even though SonoAVC required postprocessing, it decreased examination time both for patients and ultrasonographer, which helped to improve work flow in clinical practice [3,14]. 

Despite the aforementioned advantages of SonoAVC, it does take time for clinicians to shift from 2D follicular diameter to SonoAVC-generated follicular volume for follicle monitoring. For the moment the use of SonoAVC-generated d(V) and m-d values as surrogates of 2D diameter might be clinically relevant and practical, and it was the reason why we conducted this study. Moreover, we compared 2D ultrasound and SonoAVC only for the assessment of monodominant follicle in the study, because in general monodominant follicle was round or ellipsoid, but in a hyperstimulated ovary with multifollicular growth, most follicles had irregular shape due to compression by adjacent follicles. We deemed that under such an ideal situation of monofollicular growth, estimation of the consistency between two techniques might be objective and accurate. 

As a whole, Pearson correlation coefficient and ICCs calculated for manual 2D measurements and SonoAVC-generated d(V) and m-d values were both very high, predicting good concordance. This was in support with previous study [6]. When two techniques were compared in each follicle category, the results were somewhat different, Pearson correlation coefficient and ICCs lowered down regardless of follicle size, predicting moderate concordance. However, it was worthy to note that SonoAVC-generated m-d value was more comparable to 2D diameter in comparison with SonoAVC-generated d(V) value, which was evidenced by great ICCs of SonoAVC-generated m-d value and 2D diameter. 

As far as we know, the Bland-Altman analysis was extensively used to evaluate agreement between two measurement methods in numerous published studies, herein we used the Bland-Altman method for detailed analysis. In our study, the mean differences between manual 2D measurement and SonoAVC were <1 mm, and SonoAVC-generated d(V) and m-d values tended to be smaller than 2D diameter, i.e. d(V) value was less than 2D diameter by 0.82 mm on average, and m-d value was less than 2D diameter by 0.22 mm on average. In other words, SonoAVC-generated m-d value was more identical to 2D diameter than SonoAVC-generated d(V) value, which was as same as the result analyzed by ICCs. Our results were a little distinct from a study by Ata et al., they observed the SonoAVC-generated d(V) and m-d measurements were both in good agreement with conventional 2D measurements, and compared with 2D measurements, SonoAVC-generated d(V) values tended to be smaller, while SonoAVC-generated m-d values tended to be greater [7]. From the aspect of measurement error, absolute difference of <1 mm was a rather small difference in comparison with the general error of an ultrasound measurement, which was reported to be 1.2 to 3mm in 2D ultrasound [14,15]. Just as Ata et al. stated in their study, it was unlikely that the <1 mm absolute difference in the size of the follicle would affect treatment outcomes in clinic [7]. Nevertheless, our study results gave us a hint that if we used 2D ultrasound and SonoAVC interchangeably, automated SonoAVC might underestimate follicle size. 

Furthermore, we found the limits of agreements between 2D diameters and SonoAVC-generated d(V) or m-d values both became wide for large follicle size, that is to say, measurement errors of small follicles were less than that of large follicles. Indeed, it had been shown that larger follicles were associated with greater measurement error, a phenomenon existing in 2D ultrasound examination no matter by an individual observer or by several observers [15,16]. We speculated that for large follicles, measurement error within 2D ultrasound increased even by a single observer in the present study, but measurement error within SonoAVC remained stable, because SonoAVC was theoretically based on the voxel count within the identified follicle. As a result, the measurement discrepancy between two techniques enlarged for large follicles.

In summary, our study suggested that compared with manual 2D ultrasound, automated SonoAVC might underestimate follicle size, although with small absolute difference <1 mm. The absolute size of a follicle affected the consistency of two techniques.
